# Impact of early kangaroo mother care versus standard care on survival of mild-moderately unstable neonates <2000 grams: A randomised controlled trial

**DOI:** 10.1016/j.eclinm.2021.101050

**Published:** 2021-08-06

**Authors:** Helen Brotherton, Abdou Gai, Bunja Kebbeh, Yusupha Njie, Georgia Walker, Abdul K Muhammad, Saffiatou Darboe, Mamadou Jallow, Buntung Ceesay, Ahmadou Lamin Samateh, Cally J Tann, Simon Cousens, Anna Roca, Joy E Lawn

**Affiliations:** a Department of Infectious Disease Epidemiology and MARCH Centre, London School of Hygiene and Tropical Medicine (LSHTM), Keppel Street, London, UK; b MRC Unit The Gambia at LSHTM, Atlantic Road, Fajara, Gambia; c Ministry of Health, Gambia Government, Banjul, Gambia; d MRC/UVRI and LSHTM Uganda Research Unit, Nakiwogo Road, Entebbe, Uganda; e Neonatal Medicine, University College London Hospitals NHS Trust, Euston Rd, London, UK

**Keywords:** Neonate, Newborn, Premature, Survival, Mortality, Kangaroo Mother Care, Kangaroo method, Skin-to-skin contact, aPSBI, (adapted Possible Severe Bacterial Infection), aSCRIP, (adapted Stability of Cardio-respiratory in Preterm infants), bCPAP, (bubble Continuous Positive Airway Pressure), CFR, (Case-fatality rate), CI, (confidence interval), CLSI, (Clinical & Laboratory Standards Institute), CONSORT, (Consolidated Standards of Reporting Trials), CSF, (Cerebral-Spinal Fluid), DSMB, (Data Safety Monitoring Board), eKMC trial, (early Kangaroo Mother Care before Stabilisation trial), EFSTH, (Edward Francis Small Teaching Hospital), GEE, (Generalized Estimating Equation), HR, (Hazard Ratio), ICH-GCP, (International Conference on Harmonisation – Good Clinical Practice), IQR, (Inter Quartile Range), IV, (intravenous), ISO, (International organisation for standardisation), KMC, (Kangaroo mother care), LMIC, (Low and middle-income countries), LSHTM, (London School of Hygiene & Tropical Medicine), MRCG, (Medical Research Council Unit The Gambia at London School of Hygiene & Tropical Medicine), MDR, (Multi-drug resistant), NA, (not applicable), NNU, (Neonatal Unit), RCT, (Randomised controlled trial), RD, (Risk difference), RDS, (Respiratory Distress Syndrome), RR, (Risk Ratio), SAE, (Serious Adverse Event), SD, (Standard Deviation), SDG, (Sustainable Development Goal), SSA, (Sub-Saharan Africa), WHO, (World Health Organisation)

## Abstract

**Background:**

Understanding the effect of early kangaroo mother care on survival of mild-moderately unstable neonates <2000 g is a high-priority evidence gap for small and sick newborn care.

**Methods:**

This non-blinded pragmatic randomised clinical trial was conducted at the only teaching hospital in The Gambia. Eligibility criteria included weight <2000g and age 1–24 h with exclusion if stable or severely unstable. Neonates were randomly assigned to receive either standard care, including KMC once stable at >24 h after admission (control) versus KMC initiated <24 h after admission (intervention). Randomisation was stratified by weight with twins in the same arm. The primary outcome was all-cause mortality at 28 postnatal days, assessed by intention to treat analysis. Secondary outcomes included: time to death; hypothermia and stability at 24 h; breastfeeding at discharge; infections; weight gain at 28d and admission duration. The trial was prospectively registered at www.clinicaltrials.gov (NCT03555981).

**Findings:**

Recruitment occurred from 23rd May 2018 to 19th March 2020. Among 1,107 neonates screened for participation 279 were randomly assigned, 139 (42% male [*n* = 59]) to standard care and 138 (43% male [*n* = 59]) to the intervention with two participants lost to follow up and no withdrawals. The proportion dying within 28d was 24% (34/139, control) vs. 21% (29/138, intervention) (risk ratio 0·84, 95% CI 0·55 – 1·29, *p* = 0·423). There were no between-arm differences for secondary outcomes or serious adverse events (28/139 (20%) for control and 30/139 (22%) for intervention, none related). One-third of intervention neonates reverted to standard care for clinical reasons.

**Interpretation:**

The trial had low power due to halving of baseline neonatal mortality, highlighting the importance of implementing existing small and sick newborn care interventions. Further mortality effect and safety data are needed from varying low and middle-income neonatal unit contexts before changing global guidelines.

**Research in context**

**Evidence before this study**

Kangaroo mother care (KMC) is recommended by the World Health Organization (WHO) for all stable neonates ≤2000 g with the latest Cochrane review (2016) reporting 40% relative reduction in mortality at discharge or 40 – 41 weeks’ postmenstrual age, compared to standard incubator care (RR=0·60, 95% CI 0·39 – 0·92; 8 trials; 1736 neonates). This Cochrane review highlighted insufficient evidence to recommend early-onset continuous KMC before stabilisation, and recommended methodologically rigorous trials to determine the effectiveness of KMC in “unstabilised or relatively stabilised low-birth weight infants”. We searched clinicaltrials.gov and the Australian New Zealand Clinical Trials Register with the search terms “kangaroo”, “kangaroo mother care”, “kangaroo method” or “skin to skin contact AND neonate”, and identified two other trials currently ongoing or recently closed which also address this priority question (OMWaNA; clinicaltrials.gov NCT02811432 and WHO's iKMC trial; ACTRN12618001880235).

**Added value of this study**

This pragmatic, individually randomised controlled trial (*n* = 279) conducted at a Gambian level 2+ neonatal unit did not find evidence of improved survival at 28 postnatal days with early KMC versus standard care for mild-moderately unstable neonates <2000 g. Halving of inpatient case-fatality rates (48% pre-trial vs. 23% during-trial) contributed to reduced power to detect a difference in the primary outcome. There was no evidence of between-arm differences for secondary outcomes or serious adverse events however one-third of intervention neonates reverted to standard care for clinical reasons. Achieving prolonged KMC duration was challenging with barriers including absence of willing KMC providers, provision to twin pairs and need for a respectful neonatal unit environment.

**Implications of all the available evidence**

Implementation of early KMC for vulnerable unstable newborns is challenging, and studies are required in a range of neonatal care settings before this can be recommended as standard care. Implementation research is needed from perspectives of the mother/family, healthcare provider as well as health systems planning and costings data, with understanding of the KMC dose-response by risk profile a priority evidence gap. Although this trial did not show a mortality effect, findings can contribute to future meta-analyses, and demonstrate potential for substantial survival gains through improved quality small and sick newborn care.

Alt-text: Unlabelled box

## 1 Introduction

An estimated 15 million neonates are born preterm (<37 weeks gestation) annually, over 80% in Asia and Sub-Saharan Africa (SSA) [1]. Complications of preterm birth result in >1 million neonatal deaths/year [2] with the highest risk of death during the first 24 h after delivery [3]. Birth weight <2000 g is a proxy for prematurity yet this group of vulnerable neonates may also include term neonates who are small for gestational age (SGA) as well as preterm neonates with or without growth restriction. Mortality risk is greatest for preterm neonates who are also SGA [4] and all neonates <2000 g require high quality small and sick newborn care especially during the first day after birth. There is an urgent need for evidence-based interventions for neonates <2000 g in order to meet the Sustainable Development Goal 3.2 target of ≤12 neonatal deaths/1000 live births by 2030 [5].

Kangaroo mother care (KMC) is recommended as standard care for all stable neonates ≤2000 g [6]. KMC is an evidence based package of care, with key component of prolonged skin-to-skin contact between neonate and caregiver [6]. This is linked to promotion of exclusive breastmilk feeding and early hospital discharge [7]. Compared to incubator care, KMC is associated with a 36–51% reduction in mortality at discharge or at 40–41 weeks postmenstrual age [[7], [8], [9]]. There is a lack of evidence for KMC in “relatively stable or unstable” neonates [9], hence it is not currently recommended by WHO for this population [6]. This evidence-gap is a high priority [9] with several on-going or recently completed trials in SSA and South Asia [10,11].

If shown to be effective and safe, early KMC may result in a paradigm shift in hospital care of small and sick neonates, both improving outcomes and promoting family-centred care. eKMC was intended to be a pragmatic trial, aiming to assess the effect of early KMC on 28-day survival of mild-moderately unstable neonates following neonatal unit admission. As secondary objectives, we explored potential ways by which early KMC may alter preterm outcomes such as thermal control [12]; cardio-respiratory stabilisation [13]; promotion of breastfeeding [14] and avoidance of infections [9]. Secondary objectives also included safety evaluation, for which there is limited data from low and middle-income countries (LMIC).

## 2 Methods

### 2.1 Design & setting

An individually randomised superiority trial was conducted at Edward Francis Small Teaching Hospital (EFSTH), the only teaching hospital and referral Neonatal Unit (NNU) in The Gambia. Twelve percent of Gambian neonates are born preterm [1] and 29% of neonatal deaths at EFSTH are due to complications of prematurity [15] with 48% case fatality for neonates <2000 g [16]. Special newborn care (WHO Level 2+) [16] was available with oxygen via concentrators, phototherapy, pulse-oximetry for spot-checks, intravenous (IV) fluids via burettes and gastric tube feeding. Bubble CPAP (bCPAP) was introduced to the NNU in early 2018, but only became embedded in small and sick newborn care around the time of trial onset. Mechanical ventilation, surfactant, blood pressure measurement, continuous pulse oximetry monitoring and parenteral nutrition were unavailable. Running water was intermittently available, with water buckets and soap for maternal hand washing and no access to an autoclave for sterilisation of re-usable equipment. Two nurses per shift cared for up-to 80 neonates during peak periods. Continuous KMC was established as standard of care for stable neonates ≤2000 g in September 2017, provided on an eight-bed KMC unit adjacent to the NNU. An area within the NNU was identified as the “trial area”, where both control and intervention arm participants were managed.

### 2.2 Participants, screening & consent

**Fig. 1:**
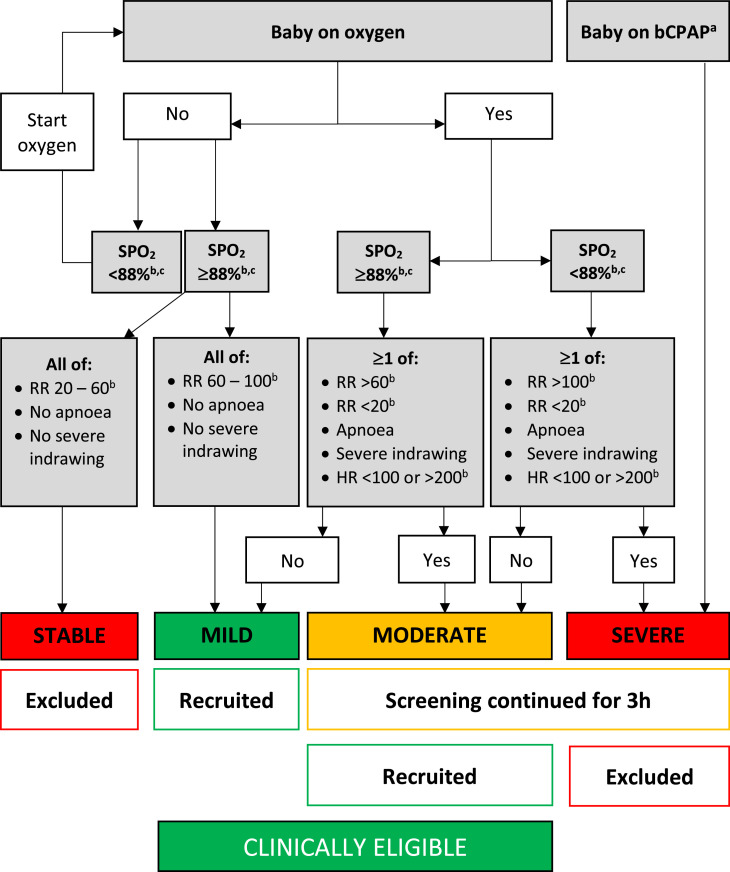
Definitions of stability used in eKMC trial. Originally published by BMC [18]. a. Criteria for starting bCPAP were: Silverman-Anderson score ≥4 with no apnoea and/or heart rate <100 bpm. b. SPO_2_, respiratory rate and heart rate were recorded every minute for a 10 min period and classified according to most frequent category of observations present for >5 min. c. Upper limit of SPO_2_ for providing oxygen therapy was 95%. Abbreviations: bCPAP= Bubble continuous positive airway pressure; RR=Respiratory rate; *h*=hours; HR=Heart rate; SPO_2_=oxygen saturation.

All admitted singleton or twin neonates weighing <2000 g and aged 1–24 h were screened for exclusion criteria, including: recruitment to another research study; triplets; major congenital malformations; severe jaundice; seizures; stable or severely unstable; absence of study bed and lack of written informed consent within 24 h of admission (Fig.S1). Presence of mother or another caregiver who was willing to provide the intervention was also required. Our target population was mild-moderately unstable neonates with severely unstable newborns excluded due to the operational challenges of providing KMC alongside resuscitation and bCPAP in our setting [17]. Stable neonates were excluded as they should already receive KMC. In the absence of validated stability scores suitable for non-intensive care settings [18], we developed pragmatic stability definitions based on clinical and cardio-respiratory observations feasible for low-resource settings, namely respiratory rate, heart rate, oxygen saturation and work of breathing (Fig. 1). Thresholds for abnormality were chosen for consistency with WHO recommended references ranges [19], with a lower oxygen saturation threshold (<88%) to avoid over classification of severe instability. Cardio-respiratory stability was assessed in potentially eligible neonates over 10 min with a Nonin™ 2500A pulse oximeter. Mildly unstable neonates were immediately recruited. Moderately and severely unstable neonates underwent repeat assessment after 3 h with exclusion of severely unstable neonates at this stage. Written informed consent was sought from the first caregiver on-site within 24 h of admission. The parent was preferred consenter but other relatives could consent with later parental assent/consent to continue participation. If consent was provided at >3 h since the last stability assessment, stability status was re-checked to avoid recruitment of neonates out-with the stability definition.

### 2.3 Randomisation, allocation and blinding

The unit of randomisation was the mother with stratification by the neonate's admission weight (<1200 g/ ≥1200 g; cut-off chosen to identify highest risk neonates). If both babies in a twin set were eligible, they were randomised to the same arm for convenience of care, according to the first eligible twins’ weight. Random permuted blocks of varying size were used and randomisation sequence was computer generated by an independent statistician. Selection bias was avoided by using sequentially numbered, opaque sealed envelopes, opened by research nurse after baseline assessment and time-stamped to identify any subversion of sequence. Due to the nature of the intervention, blinding of intervention procedures and outcome assessments wasn't possible but laboratory processes (for confirmed infections) and analyses were blinded to allocation.

### 2.4 Intervention

We defined KMC as skin-to-skin contact in the kangaroo position, with the naked (except for hat and diaper) neonate laying prone next to the caregivers’ chest in a frog-leg position with head turned sideways. A cloth wrapper [Thari design] was used to secure the neonate in KMC position, including straps tied at the sides of the infant to enable easy access [17]. Research nurses encouraged the KMC provider to start KMC immediately after allocation and to provide as close to continuous skin-to-skin contact as possible [6], aiming for >18 h/day in prolonged sessions. Beds were provided on the NNU for the exclusive use of the intervention arm caregivers. All other treatments were provided simultaneously with KMC, except for bCPAP which was provided in the high dependency area of the NNU where it was not feasible to have adult KMC beds. During breaks to pray, wash, eat etc., the neonate was placed on a non-servo controlled radiant heater or incubator in the same room. Twins received KMC from the same or different KMC providers. Clinical criteria for stopping KMC were pre-specified and included: severe instability, including need for bCPAP; apnoea needing resuscitation; widespread rash on neonate or KMC provider; severe abdominal distension; omphalitis; phototherapy; blood transfusion; seizures, and KMC provider unwilling or unavailable to provide continuous KMC. Criteria for re-starting KMC were also pre-specified (eFig.1) [17]. Neonates were transferred to the KMC unit to continue KMC once stability criteria were met and neonates tolerated enteral feeds without IV fluids for 12 h.

### 2.5 Control

Control neonates were managed in an incubator or under a radiant heater (non-servo controlled) in the same room as intervention neonates. The caregiver could touch and feed but KMC was not permitted until the neonate met stability criteria at >24 h after admission (Fig.1). Intermittent KMC (minimum 60 min skin-to-skin contact several times a day) was provided whilst the neonate was still on the neonatal unit, with continuous KMC starting after transfer to the KMC unit once stability criteria were met and neonates tolerated enteral feeds without IV fluids for 12 h.

### 2.6 Outcome measures

The primary outcome was all-cause mortality at 28 days. Secondary outcomes included: time to death; stability at 24 h, using the Stability of Cardio-Respiratory in Preterm Infants (SCRIP) score [20], modified for relevance to neonates receiving oxygen [17]; prevalence of hypothermia (axillary temperature <36·5 °C) at 24 h; proportion exclusively breastfeeding at discharge; mean daily weight gain at 28+/−5d; incidence of clinically suspected infection from age 3 to 28 days or latest follow-up, and mean duration of admission.

### 2.7 Trial procedures, including safety assessments

A detailed description of trial procedures is available in the published protocol (efig.1) [18].

To avoid preferential education of caregivers in the intervention arm, the first caregiver available on the NNU for both groups was sensitised about NNU policies, KMC provision, hand hygiene and danger signs.

Continuous pulse-oximetry monitoring was performed for trial participants during the first 24 h and continued until the neonate was stable off oxygen. Heart rate, oxygen saturation (SPO_2_), respiratory rate, blood glucose, axillary temperature, stability status, and adverse events were recorded six-hourly for the first 24 h then daily whilst on the neonatal unit. Following transfer to the KMC unit, study assessments were done on postnatal days 7, 14, 21, 28, with daily vital sign checks by EFSTH team as per standard care. Weight was measured daily from postnatal day five and length/head circumference weekly. A clinician examined participants within 24 h of enrolment and evaluated gestational age with the New Ballard score within 48 h [21].

The WHO's Possible Serious Bacterial Infection (PSBI) criteria were used to identify clinical deterioration with adaptations to increase relevance for hospitalised preterm infants by including excessive gastric aspirates, need for oxygen or bCPAP and breathing rate >80 bpm (efig.1) [17]. If ≥1 aPSBI criteria was present, neonates were assessed by a clinician for infection, which was diagnosed as per a validated nosocomial risk score for preterm neonates (age >72 h with ≥1 of apnoea, lethargy, pallor, jaundice, or hepatomegaly) [22]. Peripheral blood and CSF (if no contra-indication present) samples for culture were obtained under aseptic technique by trained clinicians if infection criteria were met (efig.1). Confirmed infection was diagnosed if suspected infection criteria were present and a known neonatal pathogen was isolated. Coagulase negative staphylococcus and bacillus species were pre-defined as non-pathogenic in this population due to the absence of indwelling catheters and lines.

Research nurses directly observed and recorded duration spent in kangaroo position for both arms, documenting timing of each KMC session, KMC provider and reason for coming out of KMC position. All other treatments were provided by EFSTH personnel according to standardised guidelines consistent with standard care and WHO recommendations for small and sick newborn care [6,19], with compliance monitored daily by trained clinicians to avoid performance bias. Guidelines included hospital discharge criteria: minimum weight 1.1 kg; >10 g/kg/day weight gain for 3 consecutive days without gastric tube feeding; both twins met weight criteria; stable vital signs and no health worker concerns; mother willing to continue KMC at home and able to attend follow-up [17]. The final study visit at postnatal age 28+/−5d was by inpatient review if admitted or scheduled follow-up at the site with re-imbursement of travel expenses and home visits for participants who did not attend.

All data were collected by Good Clinical Practice (ICH-GCP) trained personnel with three to six monthly re-training on study specific procedures. The REDCap™ electronic data entry system was used with built-in consistency checks. Double-blind standardisation assessments for gestational age and anthropometric measurements were performed to reduce inter-observer variability. Triplicate measurements of temperature and anthropometric data were obtained and cardio-respiratory parameters were measured over ten minutes to generate mean values. Calibrated equipment was used for all measurements including Seca 757 digital weighing scale (2 g gradation) and glucometers for bedside blood glucose monitoring.

### 2.8 Microbiological procedures

Blood cultures were processed within 24 h in a BACTEC 9050 BD at the MRC Unit The Gambia at LSHTM (MRCG) laboratory (ISO15189 accredited) with sub-culture by conventional methods including species identification using the API-20 system and antibiotic susceptibility testing by disc diffusion according to the Clinical and Laboratory Standards Institute (CLSI) 2018 guidelines.

### 2.9 Sample size calculation and statistical analysis

A sample size of 392 neonates (196 per arm) was chosen to provide 80% power to detect a 30% relative difference (48% vs. 34%) in mortality with a type I error rate of 5%. The baseline mortality rate of 48% was estimated from feasibility study data (56%, 14/25, in-patient case fatality rate for neonates meeting trial definitions of mild-moderate instability) assuming a 15% relative reduction related to trial implementation and was consistent with published pre-trial data [15].

Analyses were done using STATAv.16 with an intention-to-treat approach, using techniques to account for twin clustering. Between-arm differences in categorical outcomes were analysed using a generalised estimating equation (GEE) model with log link and an independent working correlation structure. A random effects model was used for continuous outcomes. Cox regression with frailty was used for time to death with right censoring of data for participants not followed up. The primary analysis was unadjusted for covariates with pre-planned analyses adjusting for twin status, weight, and gestational age. A pre-planned sub-group analysis of all outcomes was performed for weight and twin pregnancy status with an interaction term to assess for heterogeneity of treatment effects across sub-groups. All tests were two-sided and reported without adjustment for multiple testing. Missing data was low (<5%), hence complete case analysis was used.

An unblinded interim analysis was conducted by the data safety monitoring board (DSMB) after randomisation of 50% of the target sample size (*n* = 196). In March 2020 the Trial Steering Committee recommended stopping recruitment early (~70% of target sample size recruited) as the trial was recognised to now be underpowered due to reductions in baseline mortality and the COVID-19 pandemic posed an immediate risk to staff health.

### 2.10 Ethics

Approval was received from the institutional review board at LSHTM and the Gambian Government / MRCG Joint Ethics Committee. This article was prepared in accordance with CONSORT guidelines (Online-only material 1) [23].

### 2.11 Role of the funding source

The funder played no role in study design, data collection, analysis or interpretation of data, manuscript writing nor decision to submit for publication.

## 3 Results

**Fig. 2:**
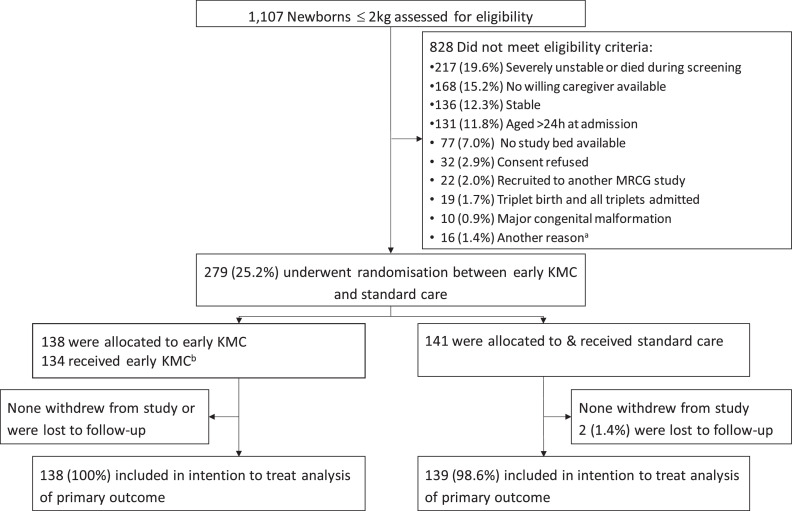
Overview of enrolment, randomisation & inclusion in intention to treat analysis of primary outcome. a. Other reasons for non-recruitment were weight ≥2000 g on trial scales (6); planned team retraining (5); seizures (3); political protests leading to temporary halt to recruitment (1) and not known (1). b. Reasons for not receiving early KMC in the intervention arm were clinical deterioration between screening and start of intervention procedures (2); no study bed available (1); no caregiver available (1). Abbreviations: *h*=hours; KMC = Kangaroo mother care.

Recruitment spanned from 23rd May 2018 to 19th March 2020 with follow-up completed by 20th April 2020. 1107 newborns were screened and 279 (25%) met eligibility criteria; 141 were allocated to receive standard care and 138 to early KMC. Among the main reasons for non-recruitment were severe instability or death during the screening process (217/1107, 19.6%), unavailability of a willing KMC provider during the first 24 h of NNU admission (168/1107, 15.2%) and limited availability of trial beds on the NNU (77/1107, 7%). There were no withdrawals and only two neonates were lost to follow-up. 277 neonates were included in the analysis of the primary outcome (Fig.2).

Our cohort consisted of mostly premature neonates (median gestational age 32 weeks in control, 33 weeks in intervention), with median admission weight <1.5 kg in both arms (1436 g control; 1459 g intervention) (Table 1). Nearly one-third (32% control vs 30% intervention) of participants were part of a twin pregnancy and 17% (both arms) of the cohort were twins with both enroled. Most neonates received oxygen therapy (89% both arms), antibiotics (87% vs. 91%) and intravenous fluids (89% vs. 83%) before allocation. 92% (control) vs. 88% (intervention) were mild or moderately unstable at the start of intervention/control procedures, with the remainder either improving or deteriorating between the end of screening and start of study procedures. There were two clinically relevant imbalances between treatment arms: (1) More neonates in the intervention arm were stable at the start of intervention/control procedures (2) Fewer neonates in the intervention arm received apnoea of prematurity prophylaxis before allocation. Baseline characteristics were otherwise balanced between arms (Table 1, eTable 1).

**Table 1:** Baseline characteristics of the intention-to-treat population.

	Standard care (*N* = 141)	early KMC (*N* = 138)
**Neonatal & perinatal characteristics**		
Male sex, N^o^ (%)	59 (42)	59 (43)
Age at admission (h), median (IQR)	2·3 (0·7 – 5)	2·3 (0·9 – 4·7)
Age at start of intervention/control^a^ (h), median (IQR)	12·8 (7·9 – 19·1)	13·6 (8·9 – 19)
Admission weight (g), median (IQR)	1436 (1180 - 1660)	1459 (1204 – 1650)
Distribution of admission weight,^b^ N^o^ (%)		
<1200 g	39 (28)	34 (25)
≥1200 g	102 (72)	104 (75)
Part of twin gestation pregnancy, N^o^ (%)	45 (32)	41 (30)
Part of twin pregnancy, both enroled, N^o^ (%)	24 (17)	24 (17)
Gestational age (weeks), median (IQR) (*n* = 271)	32 (31 - 34)	33 (31 - 34)
Distribution of gestational age, N^o^ (%)		
<28 weeks	4/135 (3)	3/136 (2)
28 – 31+6 weeks	36/135 (27)	41/136 (30)
32 – 36+6 weeks	83/135 (61)	81/136 (60)
≥37 weeks	12/135 (9)	11/136 (8)
Referral-site (EFSTH), N^o^ (%)	66 (47)	66 (48)
Health facility delivery, N^o^ (%)	121 (86)	126 (91)
Caesarean-section delivery, N^o^ (%)	27 (19)	31 (22)
Resuscitation at delivery,^c^ N^o^ (%)	10/140 (7)	5 (4)
Perinatal septic risk factors,^d^ N^o^ (%)	49/139 (35)	40/137 (29)
**Neonatal stability & management at start of intervention/control procedures**		
Stability status,^e^ N^o^ (%)		
Stable^f^	5 (4)	14 (10)
Mildly unstable	86 (61)	73 (53)
Moderately unstable	44 (31)	44 (32)
Severely unstable^f^	5 (4)	2 (1)
Axillary temperature ( °C), median (IQR)(*n* = 277)	36·6 (36·1 – 37·2)	36·6 (36·1 – 37·1)
Blood glucose (mmol/L), median (IQR)(*n* = 275)	4·1 (3·5 – 5·1)	3·8 (3·2 – 4·9)
Oxygen saturation (SPO_2_), median (IQR)(*n* = 274)	97 (96 - 98)	97 (95 - 98)
Oxygen, N^o^ (%)	125 (89)	123 (89)
Bubble CPAP, N^o^ (%)	5 (4)	2 (1)
IV antibiotics,^g^ N^o^ (%)	123 (87)	124/137 (91)
IV maintenance fluids, N^o^ (%)	125 (89)	115 (83)
IV vitamin K prophylaxis, N^o^ (%)	117 (83)	111 (80)
Apnoea of prematurity prophylaxis (IV caffeine or aminophylline), N^o^ (%)	74 (52)	57(41)

a. The start of intervention/control procedures was defined as when a trial pulse oximeter was attached to the neonate, immediately after allocation yet prior to any intervention procedures commencing.

b. Categories of admission weights as per weight cut offs used for stratification during randomisation.

c. Resuscitation at delivery with one or more of: oxygen, bag-valve-mask ventilation or chest compressions.

d. Perinatal septic risk factors included: maternal fever; maternal chorioamnionitis; offensive smelling liquor; prolonged rupture of membranes >18 h.

e. Stability definitions as per published protocol^18^ and as shown in Fig. 1.

f. Stable and severely unstable neonates were excluded during the screening phase but some eligible neonates improved or deteriorated during the consent and recruitment process, hence were stable or severely unstable when re-assessed at the start of intervention/control procedures.

g. Blood cultures were not obtained prior to antibiotic administration as they were not routinely available as part of standard care at the trial site.

Abbreviations: CPAP = Continuous positive airway pressure; EFSTH= Edward Francis Small Teaching Hospital; *g*=grams; *h* = hours; IQR = Interquartile range; IV = Intravenous; SD = standard deviation; SPO_2_ = oxygen saturation.

**Fig. 3:**
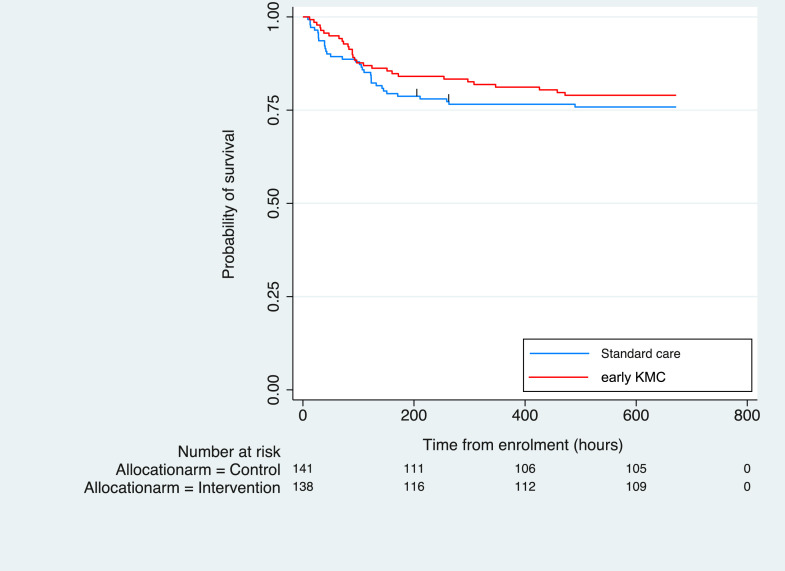
Cumulative incidence of survival over time from start of intervention/control procedures.

There was no evidence of a difference in mortality between arms in the primary intention-to-treat analysis, with 34/139 (24%) deaths in the control group versus 29/138 (21%) deaths in the intervention group (RR=0·84, 95% CI 0·55 – 1·29, Table 2) or with the survival analysis (Fig.3). Adjustment for admission weight, gestational age and twin status yielded similar results (RR=0·93, 95% CI 0·63 – 1·36, eTable 2). A sensitivity analysis excluding neonates not meeting eligibility criteria at the start of intervention/control procedures showed no evidence of between-arm effect difference (28/129 in control vs. 28/124 in intervention. RR=1·04, 95% CI 0·65 – 1·65, eTable 3).

**Table 2:** Effect of early KMC on primary and secondary outcomes.

	Standard care	Early KMC	Effect estimate *(95% CI)*	*P* value
**All-cause mortality at 28 days, N^o^(%)**	34/139 (24)	29/138 (21)	RR= 0.84 *(0·55 – 1·29)*	0·423
**Time to death (h), median (IQR)**	*N* = 139 34 deaths	*N* = 138 29 deaths	HR= 0·83 (*0·50 – 1·35)*	0·447
	98·5 (29 – 132)	90 (65 – 172)		
**aSCRIP score at 24** **h of enrolment, median, (IQR)**	*N* = 135	*N* = 134	MD −0·05 *(−0·25 – 0·16)*	0·667
	5 (4 – 6)	5 (4 – 5)		
**Hypothermia (*T*<36·5** °**C) at 24** **h of enrolment, N^o^ (%)**	55/135 (40)	51/134 (38)	RR= 0·93 *(0·69 – 1·26)*	0·654
**Exclusive breastfeeding^a^ at discharge, N^o^ (%)**	105/107 (98)	107/109 (98)	RR= 1.0 *(0·96 – 1·04)*	0·985
**Clinically suspected infection from 3 – 28 days, N^o^ (%)^b^**	21/141 (15)	28/138 (20)	RR= 1·36 *(0·81 – 2·28)*	0·240
Blood culture confirmed infection^c,d^ from 3 – 28 days, N^o^ (%)	4/141 (3)	6/138 (4)	RR= 1·53 *(0·65– 3·64)*	0·333
**Duration of admission (days), mean (SD)**	*N* = 106	*N* = 108	MD +0.3 *(−60·5 – 75·1)*	0·833
	16·3 (10·0)	16·6 (11·1)		
**Weight gain at 28d (g/day), mean (SD)**	*N* = 101	*N* = 103	MD −2·2 *(−5·28 – 0·81)*	0·150
	12·5 (12·1)	10·3 (10·1)		

Exclusively breastfeeding defined as only receiving breastmilk and no formula milk supplementation.

Defined as neonates with at least 1 suspected infection, as per protocol definition.^18^ Two neonates (one in each allocation arm) each had two discrete infection episodes.

Blood cultures were obtained from 92% (47/51) of suspected late-onset infection episodes; 95% (21/22) from control arm and 90% (26/29) from intervention arm. 21% (10/47) of blood cultures were positive with 6% (3/47) presumed contaminated samples (coagulase negative staphylococcus isolated) and no between-arm difference in mean blood volume sampled (1.1 ml (SD 0.3) in control arm versus 1.0 ml (SD 0.3) in intervention arm, *p* = 0.238, student t-test).

CSF samples were obtained from 19 neonates meeting infection criteria and all were negative after 48 h culture.

Abbreviations: CI = confidence intervals; *h* = hours; HR = Hazard ratio; IQR = Interquartile range; KMC = kangaroo mother care; MD = mean/median difference in intervention arm; RR= risk ratio; SD = standard deviation.

There was no evidence of between-arm differences in secondary outcomes, including clinically suspected infections which were relatively common (15% (21/141) of control arm versus 20% (28/138) of intervention arm. RR=1·36, 95% CI 0·81 – 2·28) and blood culture confirmed infections (RR=1·53, 95% CI 0·65– 3·64, Table 2). The ten confirmed infections were all due to gram-negative bacteria with 82% (9/11) of isolates resistant to both 3rd generation cephalosporins and gentamicin (eTable 4).

Pre-planned sub-group analyses found no evidence of between-arm differences in outcomes except for admission weight. Among neonates <1200 g, early KMC was associated with a reduction in hypothermia at 24 h (RR=0·55, 95% CI 0·33 – 0·91) with no association apparent in neonates ≥1200 g (RR= 1·29, 95% CI 0·87 – 1·91; test of interaction *p* = 0·008) (Table 3).

**Table 3:** Sub-group analysis of eKMC trial outcomes, by admission weight and twin status.

	Subgroup	N^o^ / Total N^o^ (%)		Effect size (95% CI); *P* for effect of intervention within each sub-group stratum	*P* value for test for interaction between treatment arms and sub-group strata
		Standard care (*n* = 139)	Early KMC (*n* = 138)		
**All-cause mortality at 28 days, N^o^ (%)**	Admission weight <1200 g	19/37 (51)	14/31 (45)	RR 0·88 (0·53 – 1·45); *0·614*	0·849
	Admission weight ≥1200 g	15/102 (15)	15/107 (14)	RR 0·95 (0·49 – 1·85); *0·888*	
	Singleton	25/94 (27)	21/97 (22)	RR 0·98 (0·42 – 2·29); *0·955*	0·721
	Twin pregnancy	9/45 (20)	8/41 (20)	RR 0·81 (0·49 – 1·35); *0·426*	
**Time to death, (h), median**	Admission weight <1200 g (*n* = 19; *n* = 14)	95	82	HR: 0·86 (0·43 – 1·71); *0·665*	0·888
	Admission weight ≥1200 g (*n* = 15; *n* = 15)	107	151	HR: 0·92 (0·45 – 1·89); *0·825*	
	Singleton (*n* = 25; *n* = 21)	71	109	HR: 0·75 (0·42 – 1·34); *0·334*	0·593
	Twin pregnancy (*N* = 9; *n* = 8)	123	76	HR 1.02 (0.39 – 2.64); *0.970*	
**aSCRIP score at 24** **h, mean**	Admission weight <1200 g (*n* = 36; *n* = 28)	4·6	4·6	MD 0 (−0·36 – 0·48); *0·780*	0·490
	Admission weight ≥1200 g (*n* = 99; *n* = 106)	5·1	5·0	MD −0·1 (−0·34 – 0·12); *0·358*	
	Singleton (*n* = 90; *n* = 94)	4·9	4·9	MD 0 (−0·24 – 0·25); *0·970*	0·509
	Twin pregnancy (*n* = 45; *n* = 40)	5·0	4·9	MD −0·1 (−0·51 – 0·22); *0·440*	
**Hypothermia (*T*<36·5** °**C) at 24** **h, N^o^ (%)**	Admission weight <1200 g	25/36 (691)	11/28 (39)	RR 0·54 (0·33 – 0·90); *0·018*	0·008
	Admission weight ≥1200 g	29/99 (29)	40/106 (38)	RR 1·29 (0·87 – 1·91); *0·206*	
	Singleton	41/90 (46)	30/94 (32)	RR 0·70 (0·48 – 1·02); *0·061*	0·008
	Twin pregnancy	14/45 (31)	21/40 (53)	RR 1·69 (1·0 – 2·86); *0·051*	
**Exclusive breast feeding^a^ at discharge, N^o^ (%)**	Admission weight <1200 g	18/18 (100)	17/17 (100)	RR 1·0 (0·96 – 1·05); *0·.973*	NA
	Admission weight ≥1200 g	87/89 (98)	90/92 (98)	RR 1·0 (0·96 – 1·05); *0·973*	
	Singleton	71/71 (100)	75/76 (99)	RR 1·0 (0·93–1·13); *0·605*	NA
	Twin pregnancy	34/36 (94)	32/33 (97)	RR 1·0 (0·93 – 1·13); *0·605*	
**Clinically suspected infection from 3 – 28 days, N^o^ (%)**	Admission weight <1200 g	9/37 (24)	10/31 (32)	RR 1·33 (0·62 – 2·85); 0*·470*	0·856
	Admission weight ≥1200 g	12/104 (12)	18/107 (17)	RR 1·46 (0·74 – 2·88); *0·277*	
	Singleton	16/96 (17)	22/97 (23)	RR 1·36 (0·76 – 2·43); *0·298*	0·959
	Twin pregnancy	5/45 (11)	6/41 (15)	RR 1·32 (0·43 – 4·0); *0·627*	
Blood culture confirmed infection from 3 – 28 days, N^o^ (%)	Admission weight <1200 g	0/37 (0)	1/31 (3)	RR 1·21 (0·33 – 4·41); *0·767*	NA
	Admission weight ≥1200 g	4/104 (4)	5/107 (5)	RR 1·21 (0·33 – 4·41); *0·767*	
	Singleton	2/96 (2)	3/97 (3)	RR 1·48 (0·25 – 8·71); *0·662*	0·935
	Twin pregnancy	2/45 (4)	3/41 (7)	RR 1·65 (0·29 – 9·39); *0·575*	
**Duration of admission (h), mean**	Admission weight <1200 g (*n* = 17; *n* = 17)	705·5	677·4	MD −28·1 (−174·5 – 118·4); *0·707*	0·595
	Admission weight ≥1200 g (*n* = 89; *n* = 91)	332·0	347·3	MD 15·2 (−48·4 – 78·9); *0·639*	
	Singleton (*n* = 70; *n* = 75)	410·8	404·1	MD −6·7 (−88·9 – 75·5); *0·873*	0·591
	Twin pregnancy (*n* = 36; *n* = 33)	355·2	388·2	MD 33·0 (−86·2 – 152·2); *0·588*	
**Weight gain at 28+/−5d (g/day), mean**	Admission weight <1200 g (*n* = 17; *n* = 16)	6·1	6·9	MD 0·8 (−6·64 – 8·19); *0·837*	0·369
	Admission weight ≥1200 g (*n* = 83; *n* = 88)	13·9	10·9	MD −2·9 (−6·19 – 0·33); *0·078*	
	Singleton (*n* = 65; *n* = 72)	12·9	11·2	MD −1·7 (−5·38 – 2·03); *0·377*	0·548
	Twin pregnancy (*n* = 35; *n* = 32)	11·9	8·2	MD −3·7 (−8·95 – 1·64); *0·177*	

Exclusively breastfeeding defined as only receiving breastmilk and no formula milk supplementation.

Abbreviations: CI = confidence intervals; *h* = hours; HR = Hazard ratio; IQR = Interquartile range; KMC = kangaroo mother care; MD = mean difference in intervention arm; NA = Not available; RR= risk ratio; SD = standard deviation.

21% (58/279) of participants experienced at least one clinically relevant non-fatal serious adverse event (SAE), most commonly a life threatening condition or a condition with high risk of disability (eTable 5). All SAEs were judged to be unrelated to the intervention with no between-arm differences. One third (46/138) of participants receiving early KMC met criteria to stop, at a median of 3·7d (IQR 1·6 – 6·2d), most commonly due to severe instability (16/46, 35%), isolated apnoea needing resuscitation (10/46, 22%) or needing phototherapy (8/46, 17%) (Table 4).

**Table 4:** Provision of KMC to both trial arms, with measures of intervention adherence.

	Standard care (*N* = 141)	Early KMC (*N* = 138)
Received KMC at any time during admission, N^o^ (%)	109 (77)	136 (99)
Age at starting KMC (h), median (IQR)	104·5 (73·4 – 166·1)	15·2 (10·7 – 22·0)
Started KMC within 24 h of admission, N^o^ (%)	0 (0)	119 (86)
Time from admission to first KMC (h), median (IQR)	101·1 (71.·8 – 165·1)	12 (7·4 – 17·9)
First person to provide KMC, N^o^ (%)		
Mother	98/109 (90)	73/136 (54)
Aunt	5/109 (5)	33/136 (24)
Grandmother	6/109 (6)	24/136 (18)
Other	0/109 (0)	6/136 (4)
Day 1: Duration in KMC position (h), median (IQR)	0 (0 – 0)	8.9 (5·4 – 11·7)
Day 2: Duration in KMC position (h), median (IQR)	0 (0 – 0)	7·4 (4·2 – 10·6)
Day 3: Duration in KMC position (h), median (IQR)	0 (0 – 0·1)	7·3 (2·6 – 10·5)
Day 4: Duration in KMC position (h), median (IQR)	0 (0 – 1·1)	6·8 (3·0 – 10·0)
Day 5: Duration in KMC position (h), median (IQR)	0 (0 – 3·0)	6·8 (1·8 – 9·5)
Day 6: Duration in KMC position (h), median (IQR)	0·7 (0 – 3·5)	5·8 (1·4 – 9·6)
Day 7: Duration in KMC position (h), median (IQR)	1·8 (0 – 6·0)	4·0 (0 – 9·2)
Total duration in KMC position (h), median (IQR)	21·6 (1·4 – 63·8)	66·8 (33·9 – 125·5)
Duration in KMC/day of enrolment (h), median (IQR)^a^	2·1 (0·2 – 3·7)	6·7 (4·3 – 8·5)
Days that ≥1 h of KMC provided, median (IQR)	5 (1 – 10)	9.5 (5 – 16)
Proportion discontinuing intervention, N^o^ (%)	NA	46 (33·3)
Reason for discontinuation of intervention, N^o^ (%)		
Severely unstable^b^	NA	16/46 (35)
Isolated apnoea needing resuscitation	NA	10/46 (22)
Severe jaundice	NA	8/46 (17)
Recurrent hypoglycaemia	NA	2/46 (4)
Severe abdominal distension	NA	2/46 (4)
Other^c^	NA	8/46 (17)
Age at stopping intervention (days), median (IQR)	NA	3·7 (1·6 – 6·2)
Proportion re-starting KMC once stability criteria met		22/46 (48)

a. 11% (15/138) of the intervention arm and 0.7% (1/141) of the control arm spent >10 h/d in KMC position from enrolment to discharge or last study visit if admitted beyond 28d of age.

a. Severe instability defined as per protocol criteria^18^ and in Fig. 1.

b. Other reasons for discontinuation of the intervention were: seizures; omphalitis; neonatal skin infection; maternal skin infection; blood transfusion; non-severe presentation of infection; aspiration of milk; died (*n* = 1 each).

Abbreviations: CI = confidence intervals; *h* = hours; IQR = Interquartile range; KMC = kangaroo mother care; NA = Not available.

99% (136/138) of neonates in the intervention arm received KMC with 86% (119/138) starting KMC within 24 h of admission (median age 15·2 h). The longest time in the KMC position was on day one (median 8·9 h/day), reducing to between 4 – 7.4 h/day from day two (Table 4). Over three-quarters (77%, 109/141) of control participants received KMC during admission, none within the first 24 h and median 4·4 days old at initiation (Table 4, eFig.2).

There was no evidence of differences in the proportions of participants receiving concomitant oxygen (97% in control vs. 95% in intervention), bCPAP (13% vs. 14%), ampicillin and gentamicin (99% in both arms), gastric tube feeding (86% vs. 91%) and apnoea of prematurity prophylaxis (82% vs. 75%) during hospital stay. However, control participants received more meropenem (4 vs 0; *p* = 0·046) and less cefuroxime than intervention participants (0 vs 5; *p* = 0·023) (eTable 6).

## 4 Discussion

This randomised trial in a Gambian level 2+ neonatal unit did not provide evidence that early KMC for mild-moderately unstable neonates results in a mortality reduction compared with standard care. A halving of in-patient case-fatality rates (CFR) (48% pre-trial [15] vs. 23% during the trial) contributed to reduced power (~30%) to detect a 30% between-arm difference and the target sample size was not achieved. The median duration spent in KMC position for intervention participants was 6.7 h/day, reflecting known challenges in achieving prolonged KMC duration [24] and possibly contributing to the lack of effect. Secondary outcomes showed no between-arm differences, except for reduced hypothermia in neonates <1200 g.

The halving of baseline CFR in our cohort was likely influenced by both improvements to small and sick newborn care during the trial preparation phase (e.g. KMC implementation for stable newborns, increasing use of bCPAP) and enhanced clinical monitoring necessary for ethical trial conduct [25], protocol compliance and avoidance of performance bias.

Despite caregiver education and efforts to promote compliance, intervention neonates spent less than the recommended 18 h/day^6^ in the kangaroo position, with median 6.7 h/day. The minimum threshold of KMC exposure for a mortality reduction to be achieved is not known [6,9] and mortality reductions are reported with >20 h/day[9] and >22 h/day [8]. The iKMC trial reported an increased risk of death for neonates receiving <10 h/day of skin-to-skin contact, but this may have been confounded by medical issues precluding longer durations [26]. Despite known benefits, ≥18 h/day KMC for stable neonates is often not achieved [24,27]. Promoting early KMC for unstable neonates for prolonged periods is even more challenging [28]. The longest duration spent in KMC position for our intervention neonates was during the first 24 h of trial participation (median 8.9 h/day), when neonates were still receiving oxygen, IV fluids and undergoing 6-hrly trial assessments. There-after, the daily duration reduced to between 4 h/day and 7.4 h/day. The iKMC trial achieved KMC duration of 16.9 h/day on the neonatal unit, with maternal support from dedicated study personnel [26]. Providing intensive health worker support for KMC was not possible within our trial as both research and hospital personnel had high workloads and multiple responsibilities with low nurse to patient ratios. KMC sessions were interrupted for medical procedures, routine neonatal cares (feeding, including expression of mother's milk) and for the KMC provider to rest, eat, pray and bathe. Other challenges to providing prolonged KMC duration included mothers being absent due to maternal illness, post-caesarean section or delivery at another health facility. This is reflected in nearly half of our intervention arm receiving first KMC contact from a female relative (aunt/grandmother) and highlights the importance of family support for KMC [29]. The high proportion of twins (17% of our cohort were twins both enroled) may have also affected provision of prolonged KMC, due to the reticence of KMC providers to perform KMC with unstable twins simultaneously. The introduction of adult beds onto the NNU to enable continuous KMC was an important operational challenge, requiring re-organisation of patient flow, consideration of the infection prevention control implications and need to provide a respectful environment.

Our findings are in contrast to those from a small Ethiopian single centre trial reporting a 40% mortality reduction with KMC at < 24 h after delivery [30]. Detailed information on screening, randomisation and baseline stability were not reported, hence we cannot adequately compare populations and study design. However, a lower proportion received oxygen (~35% vs 89% in eKMC trial) and IV fluids (55% vs 86% in eKMC trial), suggesting a more stable cohort in the Ethiopian trial. The iKMC trial recently reported a 25% relative reduction in 28-day mortality (RR 0.75, 95% CI 0.64 –0.89, *p* = 0.001) with immediate KMC, started at 1.3 h after birth [27]. This multicentre trial recruited from five tertiary hospitals in India and Africa and identified statistically significant 28-day mortality reductions in the sub-groups of neonates 1.5 – 1.799 kg, singletons and those recruited at the Indian site [26]. An important difference with our trial was that iKMC sites had a higher level and quality of newborn care (WHO level 3), indicated by the lower control arm mortality rate of 15.7% and lower prevalence of hypothermia (10% vs 38–40% in our cohort) [26]. Information about the stability status of iKMC participants at baseline is not provided, hence we cannot make direct comparisons with our cohort. Another important difference was our inclusion of extremely low birth weight (<1 kg) neonates, comprising 11% of our cohort and not represented in the iKMC trial [26]. This may have further reduced intervention effects in our study due to the high risk of surfactant deficiency and our inability to simultaneously provide bCPAP and KMC.

In contrast to evidence that KMC improves stability scores at postnatal 6 h [8,13], we found no evidence of a difference at 24 h of enrolment. More detailed analyses are planned to compare with existing evidence that KMC positively regulates respiratory stability [8]. Our finding that KMC reduces hypothermia in neonates <1200 g is consistent with existing evidence [8,9] and has clinical significance for this population at greatest risk of hypothermia. Both arms were admitted for 16 days with no difference between arms. This is similar to the duration of stay of approximately two weeks reported by iKMC [26] and likely reflects the lack of effect of early KMC on weight gain and breastfeeding in our cohort, which were the main criteria for discharge.

The absence of effect of KMC on infections contrasts with previous meta-analyses reporting a 65% reduction in nosocomial infection [9] and 50% reduction in severe infection [8] with KMC in stable newborns. However, previous KMC trials used varied clinical infection definitions [8,9,26] and we are the first to report clear apriori clinical infection definitions combined with microbiologically confirmed infections and low-risk of detection bias. We found no evidence that KMC reduces infections, consistent with three previous studies reporting culture-confirmed infections which were included in the most recent Cochrane review [9]. The iKMC trial reported a 18% reduction in suspected sepsis with immediate KMC (RR 0.82, 95% CI 0.73 – 0.93) but used a non-validated non-specific clinical definition without microbiological confirmation. Two of the iKMC sites admitted control and intervention neonates to different NICUs, with newly built Mother-NICUs for the intervention arm [26]. Thus, the possibility of varying environmental exposures for nosocomial infections cannot be excluded and detection bias is also a risk. As for many LMIC neonatal units, infection prevention is a major challenge at EFSTH, with recent endemic *Burkholderia cepacia* and epidemic multi-drug resistant (MDR) *Klebsiella pneumoniae* outbreaks with contaminated intravenous fluids and antibiotics implicated in transmission [30,31]. This is consistent with the predominance of MDR-gram negative bacteria causing invasive infections in our cohort and, although we cannot comment on acquisition of invasive isolates, this warrants further study. It is possible that the effect of KMC on reducing infection risk may vary depending on the nosocomial context of the setting. Environmental exposures such as contaminated fluids and antibiotics are an infection risk regardless of when KMC is started and strengthening of infection prevention control procedures, including promotion of hand hygiene for KMC providers and health workers, should be ensured before KMC implementation and provision.

We cannot fully determine the safety of early KMC due to having low power for mortality, however we observed that it can be provided safely if continuous pulse-oximetry monitoring and targeted caregiver education are also in place. Pulse-oximetry monitoring should be part of safe oxygen provision for all neonates [6], yet is inconsistently available in many LMIC settings and was not widely available at the site prior to trial implementation. We advise caution to extrapolating our safety findings to settings without continuous pulse-oximetry monitoring, as one-third of the intervention group stopped KMC due to clinical deterioration and prompt detection of life-threatening conditions is essential. We recommend that neonates who are receiving KMC at the same time as oxygen should be continuously monitored with pulse oximetry.

eKMC is one of the first RCTs addressing the priority question of the mortality effect of early KMC in unstable neonates. Our results are generalisable to LMIC level 2/2+ neonatal units providing oxygen and continuous pulse-oximetry which have a high risk of nosocomial infection. As per Cochrane recommendations, we report clear definitions of eligibility and stability [9] and used a clinical infection definition based on a validated clinical score for preterm neonates [22] as well as reporting blood culture confirmed infections. We achieved high levels of protocol compliance for timing of KMC initiation, with a large between-group gap, but did not achieve targets of >18 h/day. KMC trials have some inherent limitations such as the inability to blind the intervention. Despite meticulous screening and allocation methods, there were minor differences in baseline stability between arms but these are probably due to chance and are not likely to have affected our results, as shown by the sensitivity analysis. We minimised performance bias by managing both arms in the same environment, implementing a standardised guideline and ensuring comparable between-group education. We had limitations in our data collection methods for KMC duration due to small size of our research team and possible under-estimation of KMC delivered due to high work-load and competing responsibilities. Accurate and validated methods of measuring KMC duration is a research gap with relevance for both routine health management information systems [24] and other KMC trials.

Due to improvements in survival and cessation of the trial before achieving the intended sample size, our trial had low power for the primary outcome but was adequately powered for some secondary outcomes. Our results may contribute to future meta-analyses and give safety and implementation insights into early KMC use in unstable neonates. Our methods and outcomes are purposefully similar to other, larger trials in LMIC [10].

More data about effectiveness and safety of early KMC is needed from settings with similar contexts of care. Understanding the minimum KMC duration needed for mortality effect is a key gap. Further research into infection prevention effects of KMC is needed with standardised definitions, microbiological and genomic analysis, including effects on neonatal microbiome and MDR-gram negative bacteria carriage. Implementation of KMC for any stability level is challenging and we urgently need more insights into how to promote prolonged KMC for stable and unstable neonates from health systems, health worker and mother/family perspectives, including economic evaluations.

The halving of mortality during the trial implementation period highlights the substantial survival gains possible with higher quality implementation of currently recommended small and sick newborn care. Due to low power, we cannot draw definitive conclusions about the mortality effects of early KMC in unstable neonates. However, our results may contribute to meta-analyses and provide important safety and implementation insights into the use of early KMC in unstable neonates on level 2/2+ special care neonatal units. Larger trials from similar settings are needed before policy and programmatic change can be recommended.

## Contributors

HB and JEL conceived of the research question, obtained the funding and designed the trial with input from AR, CT and SC. The trial was implemented by HB, AG, BK, GW and ALS with data collection by AG, BK and GW. SD, MJ and BC were responsible for processing and reporting of microbiological data. SD was the laboratory analytical project manager. YN was responsible for data cleaning and verification. HB had access to the raw data and performed the statistical analysis with AKM. HB developed the figures and tables with exception of the supplementary heat map which was developed by AKM. HB wrote the original draft of the manuscript with critical revision for important intellectual content from JEL, AR, CT and SC. All authors contributed to the final version of the manuscript, approved the final manuscript as submitted and agree to be accountable for all aspects of the work.

## Declaration of Competing Interest

We declare no competing interests.

## Appendix Supplementary materials

Image, application 1

Image, application 2
