# Supplementary material for: Impact of early kangaroo mother care versus standard care on survival of mild-moderately unstable neonates <2000 grams: A randomised controlled trial
Source: eClinicalMedicine. 2021 Aug 6;39:101050. doi: 10.1016/j.eclinm.2021.101050 (PMC8358420; doi:10.1016/j.eclinm.2021.101050)
Supplement: Supplementary file 1 [file mmc1.docx]

# **Supplementary materials**

1. eKMC trial protocol (V4.0, 18^th^ Mar 2019)
2. Statistical analysis plan for eKMC trial
3. CONSORT checklist
4. Annex 1: Supplementary tables & figures

This annex has been provided by the authors to give readers additional information about the trial.

eTable 1. Additional baseline characteristics for intention-to-treat population

eTable 2. Secondary analysis for eKMC primary and secondary outcomes, adjusted for twin status, admission weight and gestational age

eTable 3. Sensitivity analysis excluding participants not meeting eligibility criteria at start of intervention/control procedures

eTable 4. Overview of neonates with blood-culture confirmed infections from 3d – 28d, including outcome and phenotypic MDR status of bacterial isolates

eTable 5. Non-fatal Serious Adverse Events during eKMC trial, for intention-to-treat population

eTable 6. Concomitant medications received by intention-to-treat population during hospitalisation

eFigure 1. Overview of eligibility criteria, study procedures and key definitions for eKMC trial

eFigure 2. Duration (minutes) spent in kangaroo position, by allocation arm and day of enrolment
