# Supplementary material for: Impact of early kangaroo mother care versus standard care on survival of mild-moderately unstable neonates <2000 grams: A randomised controlled trial
Source: eClinicalMedicine. 2021 Aug 6;39:101050. doi: 10.1016/j.eclinm.2021.101050 (PMC8358420; doi:10.1016/j.eclinm.2021.101050)
Supplement: Supplementary file 2 [file mmc2.docx]

**eTable 1. Additional baseline characteristics for intention-to-treat population**

|  | **Standard care**  **(n=141)** | **KMC before stabilisation**  **(n=138)** |
| --- | --- | --- |
| **Neonatal** | | |
| Admission length (cm) median (IQR)^a^ | 40·1 (37·3 – 42·3) | 40·1 (37·6 – 42·0) |
| Admission head circumference (cm) median^b^ (IQR) | 28·5 (26·3 – 29·9) | 28·2 (26·8 – 29·5) |
| NMR2000 score,^c^ median (IQR) | 17·2 (14·5 – 19·5) | 17·6 (14·7 – 19·5)^d^ |
| Hypothermia (axillary temp <36.5^o^C), N^o^ (%) | 54 (38%) | 46/137 (34%) |
| Hyperthermia (axillary temp >37.5 ^o^C), N^o^ (%) | 15 (11%) | 19/137 (14%) |
| Hypoglycaemia (<2.6 mmol/L), N^o^ (%) | 4 (3%) | 8/134 (6%) |
| Hyperglycaemia (>6.9 mmol/L), N^o^ (%) | 19 (13%) | 13/134 (10%) |
| Respiratory rate (bpm), median (IQR)^e^ | 56·1 (47·8 – 66·7) | 57·1 (47 – 65·9) |
| Heart rate (bpm), median (IQR)^f^ | 138·3 (130 - 149) | 138·4 (125 – 148·3) |
| Bag valve mask ventilation, N^o^ ( %) | 1 (1%) | 4 (3%) |
| Chest compressions, N^o^ ( %) | 3 (2%) | 3 (2%) |
| IV Fluid bolus, N^o^ ( %) | 9/140 (6%) | 5 (4%) |
| Gastric tube in-situ, N^o^ ( %) | 85 (60%) | 84 (61%) |
| Expressed breast milk given, N^o^ ( %) | 3 (2%) | 7 (5%) |
| Blood transfusion, N^o^ ( %) | 1 (1%) | 2 (2%) |
| **Mother** | | |
| Maternal age (years) median (IQR) | 26 (21 - 31) | 25 (20 - 30) |
| English illiterate, N^o^ (%) | 88/140 (63%) | 77/137 (56%) |
| Married, N^o^ (%) | 132 (94%) | 126 (91%) |
| Level of education, N^o^ (%) |  |  |
| No formal education | 42/138 (30%) | 42/137 (31%) |
| Islamic school | 26/138 (19%) | 30/137 (22%) |
| Primary school | 21/138 (15%) | 13/137 (9%) |
| Secondary school | 42/138 (30%) | 43/137 (32%) |
| College/university | 7/138 (5%) | 9/137 (7%) |
| Employment, N^o^ (%) | | |
| No formal employment^g^ | 112/139 (81%) | 118/137 (86%) |
| Domestic service | 1/139 (1%) | 6/137 (4%) |
| Unskilled manual | 7/139 (5%) | 3/137 (2%) |
| Skilled manual | 8/139 (5%) | 3/137 (2%) |
| Sales & service or clerical | 5/139 (4%) | 0/137 (0%) |
| Professional/managerial/technical | 7/139 (5%) | 7/137 (5%) |
| Urban residence, N^o^ (%) | 113 (80%) | 107/137 (78%) |
| Parity, N^o^  (%) | | |
| Primiparous | 38 (27%) | 50/136 (37%) |
| 2 – 7 | 99 (70%) | 78/136 (57%) |
| >7 | 4 (3%) | 8/136 (6%) |
| Maternal co-morbidities, N^o^ (%) | | |
| Hypertension | 22 (16%) | 32 (23%) |
| Anemia (<10g/dl) during 2^nd^ or 3^rd^ trimester | 4 (3%) | 5 (4%) |
| HIV | 2 (1%) | 2 (1%) |
| Infection needing antibiotics in 3^rd^ trimester | 3 (2%) | 1 (1%) |
| Bleeding needing blood transfusion | 3 (2%) | 0 (0) |
| Eclampsia | 1 (1%) | 1 (1%) |
| Diabetes | 1 (1%) | 1 (1%) |
| Antenatal clinic visits, N^o^ (%) | | |
| No visits | 12/140 (9%) | 4/136 (3%) |
| 1 visit | 18/140 (13%) | 19/136 (14%) |
| 2 – 4 visits | 87/140 (62%) | 83/136 (61%) |
| 5 or more antenatal clinic visits | 23/140 (16%) | 30/136 (22%) |
| Tetanus vaccine (at least 1 dose), N^o^ (%) | 102 (72%) | 112 (81%) |
| Malaria IPT (at least 1 dose), N^o^ (%) | 126 (89%) | 121 (88%) |
| **Perinatal** | | |
| Delivery lasted >24h, N^o^ (%) | 28/140 (20%) | 30 (22%) |
| Apgar score at 5 minutes (median)(IQR)^h^ | 9 (7 - 10) | 10 (7 - 10) |
| Maternal antibiotics within 7 days before birth, N^o^ (%) | 14/140 (10%) | 17 (12%) |
| Any septic risk factor, N^o^ (%) | 50/139 (36%) | 40/137 (29%) |
| PROM >18h | 24/139 (17%) | 18/137 (13%) |
| Maternal fever within 48h of delivery | 30/139 (22%) | 27/137 (20%) |
| Foul smelling liquor | 9/138 (7%) | 6/137 (4%) |
| Chorioamnionitis | 0/140 (0) | 1/137 (1%) |
| Cord hygienically cut,^i^ N^o^ (%) | 98 (70%) | 95 (69%) |
| Cord hygienically clamped,^j^ N^o^ (%) | 133 (94%) | 132 (96%) |
| Bathed after delivery, N^o^ (%) | 1 (1%) | 1 (1%) |

a. Admission length missing for 7 participants, n = 138 for control arm and n=134 for intervention arm

b. Admission head circumference missing for 4 participants, n=139 for control arm and n=136 for intervention

c. NMR score is a validated mortality risk score including the following parameters: Birthweight; oxygen saturation level and highest level of respiratory support needed^.19^

d. NMR score missing for 5 participants in the intervention arm

e. Baseline respiratory rate missing for 5 participants, n=140 for control arm and n=134 for intervention arm

f. Baseline heart rate missing for 6 participants, n=139 for control arm and n=134 for intervention arm

g. Subsistence farming and informal childcare / house-work or informal childcare were classified as being not formally employed.

h. Apgar score at 5-minutes missing for 166 participants, n=48 for control arm and n=65 for intervention arm

i. Hygienic cord cutting defined as cutting with clean razor or scalpel.

j. Hygienic cord clamping defined as new plastic cord clamp used.

Abbreviations: HIV = Human Immunodeficiency Virus; I = Intervention; IPT = Intermittent prophylactic treatment; IQR = Interquartile range; IV = Intravenous; NMR2000 = neonatal mortality risk 2000 score; PROM = Prolonged rupture of membranes.

**eTable 2. Secondary analysis for eKMC primary and secondary outcomes, adjusted for twin status, admission weight and gestational age**

|  | Standard care | KMC before stabilisation | Effect size  (95% CI) | P value |
| --- | --- | --- | --- | --- |
| All-cause mortality at 28 days, N^o^(%) | 30/133 (23%) | 28/136 (21%) | RR= 0·93  (0·63 – 1·36) | 0·699 |
| Time to death (h), median (IQR) | N=30 | N=28 | HR= 0·91  (0·54 – 1·53) | 0·716 |
|  | 106 (39 – 142) | 92 (68 – 213) |  |  |
| aSCRIP score at 24h of enrolment, median, (IQR) | N=133 | N=133 | MD – 0·06  (-0·26 – 0·14) | 0·541 |
|  | 5 (4 – 6) | 5 (4 – 5) |  |  |
| Hypothermia (T<36·5 ^o^C) at 24h of enrolment, N^o^ (%) | 53/133 (40%) | 51/133 (38%) | RR= 0·92  (0·69 – 1·22) | 0·574 |
| Exclusive breastfeeding^a^ at discharge, N^o^ (%) | 105/107 (98%) | 107/109 (98%) | RR= 0·99  (0·96 – 1·01) | 0·381 |
| Clinically suspected infection from 3 – 28 days, N^o^ (%) | 21/135 (16%) | 28/136 (21%) | RR= 1·29  (0·79 – 2·12) | 0·304 |
| Duration of admission (days), mean (SD) | N=104 | N=107 | MD 7·5  (-49·0 – 64·0) | 0·796 |
|  | 386·4 (240·7) | 400·7 (266·8) |  |  |
| Weight gain at 28d (g/day), mean (SD) | N=99 | N=103 | MD -2·3  (-5·2 – 0·63) | 0·125 |
|  | 12·6 (12·2) | 10·2 (10·2) |  |  |

1. Exclusively breastfeeding defined as only receiving breastmilk and no formula milk supplementation

Abbreviations: CI = confidence intervals; HR = Hazard ratio; MD = mean/median difference in intervention arm; RR= risk ratio; SD = standard deviation

**eTable 3. Sensitivity analysis of eKMC outcomes excluding participants not meeting eligibility criteria at start of intervention/control procedures^a^**

|  | Standard care | KMC before stabilization | Effect size  (95% CI) | P value |
| --- | --- | --- | --- | --- |
| All-cause mortality at 28 days, N^o^(%) | 28/129 (24%) | 28/124 (23%) | RR= 1·04  (0·65 – 1·65) | 0·867 |
| Time to death (h), median (IQR) | N=28 | N=28 | HR= 1·02  (0·61 –1·73) | 0·933 |
|  | 98·5  (34 – 128) | 91·5  (68 – 213) |  |  |
| aSCRIP score at 24h of enrolment, median, (IQR) | N=125 | N=121 | MD -0·1  (-0·32 – 0·09) | 0·264 |
|  | 5 (4 – 6) | 5 (4 – 5) |  |  |
| Hypothermia (T<36·5 ^o^C) at 24h of enrolment, N^o^ (%) | 49/125 (39%) | 46/121 (38%) | RR= 0·97  (0·71 – 1·33) | 0·849 |
| Exclusive breastfeeding at discharge,^b^ N^o^ (%) | 99/101 (98%) | 94/96 (98%) | RR= 1·0  (0·96 – 1·04) | 0·978 |
| Clinically suspected infection from 3 – 28 days, N^o^ (%) | 20/129 (16%) | 28/124 (23%) | RR= 1·46  (0·87 – 2·45) | 0·156 |
| Duration of admission (days), mean (SD) | N=100 | N=95 | MD 0·5  (-61·5 – 83·5) | 0·767 |
|  | 16·2 (10·2) | 17·0 (11·4) |  |  |
| Weight gain at 28d (g/day), mean (SD) | N=97 | N=92 | MD= -1·9  (-5·0 – 1·28) | 0·244 |
|  | 12·2 (11·8) | 10·3 (10·3) |  |  |

1. Total of 24 neonates were excluded from this analysis. This included 2 neonates who were recruited in error: One aged >24h and one who was incorrectly classified as being moderately unstable during screening but was severely unstable on clinician checking of stability status. 22 neonates were excluded who met stability criteria at time of screening but either improved or deteriorated during the recruitment period and hence no longer met eligibility criteria at the start of intervention/control procedures (stable=16; severely unstabl =6).
2. Exclusively breastfeeding defined as only receiving breastmilk and no formula milk supplementation

Abbreviations: CI = confidence intervals; HR = Hazard ratio; MD = mean/median difference in intervention arm; RR= risk ratio; SD = Standard deviation.

**eTable 4.** Overview of neonates with blood-culture confirmed infections from 3d – 28d, including outcome and phenotypic MDR status of bacterial isolates

| **Sex** | **Age at illness onset (days)** | **Gest. age (wks)** | **Admission weight (g)** | **Isolate (MDR)^a^** | **Antibiotic susceptibility**  **(S/R)** | | | **Outcome** |
| --- | --- | --- | --- | --- | --- | --- | --- | --- |
|  |  |  |  |  | **3^rd^ gen ceph** | **Gent** | **Cipro** |  |
| **Intervention arm** | | | | | | | | |
| Female | 3.2 | 34 | 1730 | Burkholderia cepacia | S | R | R | Recovered |
| Male | 4.7 | 36 | 1634 | Burkholderia cepacia | S | R | R | Recovered |
| Male | 3.5 | 32 | 1606 | Shigella spp (MDR) | R | R | R | Died |
| Female | 3.8 | 29 | 1032 | Acinetobacter spp (MDR) | R | R | R | Died |
| Female | 12.9 | 28 | 1500 | Klebsiella pneumoniae^b^ (MDR) | R | S | R | Died |
|  |  |  |  | Enterobacter spp^b^ (MDR) | R | R | R |  |
| Male | 5.6 | 32 | 1544 | Shigella spp (MDR) | R | R | S | Recovered |
| **Control arm** | | | | | | | | |
| Female | 4.8 | 32 | 1366 | Shigella spp (MDR) | R | R | R | Died |
| Male | 4.6 | 30 | 1468 | Pseudomonas spp (MDR) | R | R | R | Died |
| Male | 11.7 | 32 | 1202 | Pseudomonas spp | R | S | S | Died |
| Male | 8.1 | 34 | 1434 | Raoultella Ornithinolytica (MDR) | R | R | R | Died |

1. Phenotypic MDR defined as resistance to at least one agent in >3 different classes of antimicrobial agents with resistance determined as per CLSI 2018 guidelines
2. Mixed growth from one participant

Abbreviations: MDR = multi-drug resistant; R = Resistant; S = sensitive; SD = standard deviation); spp = species

**eTable 5. Non-fatal Serious Adverse Events (SAE) during eKMC trial for intention-to-treat population**

| Non-fatal SAE type^a^ | Standard care  N=141 | KMC before stabilisation  N=138 | Total  N=279 |
| --- | --- | --- | --- |
| Life threatening^b^, N^o^ (%) | 11 (8%) | 13 (9%) | 24 (9%) |
| Risk of disability^c^, N^o^ (%) | 7 (5%) | 6 (4%) | 13 (5%) |
| Prolonged hospitalisation >28 days, N^o^ (%) | 5 (4%) | 7 (5%) | 12 (4%) |
| Hospital re-admission within 28 days, N^o^ (%) | 5 (4%) | 4 (3%) | 9 (3%) |
| Total non-fatal SAEs / study population, N^o^ (%) | 28 (20%) | 30 (22%) | 58 (21%) |

a. Classified according to final SAE report for all participants.

b. Life threatening SAEs defined as apnoea needing resuscitation, severe instability as per protocol definition and any other life threatening situation as assessed by a clinician.

c. Defined as any condition placing the participant at increased risk of permanent or temporary disability, such as suspected or confirmed meningitis, jaundice needing treatment or acquired hydrocephalus requiring medical or surgical intervention.

Abbreviations: KMC = Kangaroo mother care; SAE = Serious adverse event

**eTable 6. Concomitant treatments received by intention-to-treat population during hospitalisation**

|  | Standard care  (n=141) | KMC before stabilisation  (n=138) | P value^a^ (95% CI) |
| --- | --- | --- | --- |
| Oxygen, N^o^ (%) | 137 (97%) | 131 (95%) | 0·337 (-0·2 – 0·07) |
| bCPAP, N^o^ (%) | 18 (13%) | 20 (14%) | 0·674 (-0·10 – 0·06) |
| >1 episode of bag-valve-mask ventilation, N^o^ (%) | 36 (26%) | 32 (23%) | 0·649 (-0·08 – 0·12) |
| >1 episode of chest compressions, N^o^ (%) | 30 (21%) | 29 (21%) | 0·957 (-0·09 – 0·10) |
| Gastric tube feeding, N^o^ (%) | 121 (86%) | 126 (91%) | 0·103 (-0·13 – 0·01)_ |
| Maintenance IV fluids, N^o^ (%) | 128 (91%) | 126 (91%) | 0·878 (-0·07 – 0·06) |
| >1 10% Dextrose bolus (IV) for hypoglycaemia, N^o^ (%) | 50/136 (37%) | 56/135 (41%) | 0·426 (-0·16 – 0·07) |
| Mothers’ expressed breast milk, N^o^ (%) | 124 (88%) | 124 (90%) | 0·611 (-0·09 – 0·05) |
| Formula milk, N^o^ (%) | 24 (17%) | 28 (20%) | 0·483 (-0·12 – 0·06) |
| Phototherapy, N^o^ (%) | 8 (6%) | 9 (7%) | 0·767 (-0·06 – 0·05) |
| Blood transfusion, N^o^ (%) | 11 (8%) | 10 (7%) | 0·874 (-0·06 – 0·07) |
| IV Ampicillin, N^o^ (%) | 140 (99%) | 137 (99%) | 0·988 (-0·02 – 0·02) |
| Number of ampicillin doses, mean (SD) | 8.4 (4.2) | 9.7 (7.9) | 0·076 (-2·8 – 0·14) |
| IV Gentamicin, N^o^ (%) | 140 (99%) | 137 (99%) | 0·988 (-0·02 – 0·02) |
| Number of gentamicin doses, mean (SD) | 4.8 (2.8) | 5.3 (3.2) | 0·208 (-1·16 – 0·25) |
| IV Ceftriaxone, N^o^ (%) | 33 (24%) | 30 (22%) | 0·715 (-0·08 – 0·12) |
| Number of ceftriaxone doses, mean (SD) | 5.6 (4.0) | 5.7 (4.1) | 0·904 (-2·16 – 1·92) |
| IV Flucloxacillin, N^o^ (%) | 6 (4%) | 6 (4%) | 0·970 (-0·05 – 0·05) |
| IV Ciprofloxacin, N^o^ (%) | 2 (1%) | 4 (3%) | 0·394 (-0·05 – 0·02) |
| IV Metronidazole, N^o^ (%) | 11(8%) | 7/137 (5%) | 0·362 (-0·03 – 0·08) |
| IV Meropenem, N^o^ (%) | 4 (3%) | 0 (0) | 0·046 (0·001 – 0·06) |
| IV Co-amoxiclav, N^o^ (%) | 11 (8%) | 9 (7%) | 0·679 (-0·05 – 0·07) |
| IV Piperacillin-Tazobactam, N^o^ (%) | 3 (2%) | 0 (0) | 0·085 (-0·003 – 0·05) |
| IV Cefuroxime, N^o^ (%) | 0 (0) | 5 (4%) | 0·023 (-0·07 - -0·01) |
| Other antibiotic, N^o^ (%) | 3 (2%) | 0 (0) | 0·085 (-0·003 – 0·05) |
| IV Vitamin K prophylaxis, N^o^ (%) | 120 (85%) | 123 (89%) | 0·316 (-0·12 – 0·04) |
| IV Vitamin K treatment, N^o^ (%) | 10 (7%) | 8 (6%) | 0·660 (-0·04 – 0·07) |
| IV Caffeine citrate prophylaxis, N^o^ (%) | 53/140 (38%) | 57/137 (42%) | 0·524 (-0·15 – 0·08) |
| Number of caffeine doses, mean (SD) | 6 (4.2%) | 7.4 (4.0) | 0·091 (-2·93 – 0·22) |
| IV Aminophylline prophylaxis, N^o^ (%) | 62 (44%) | 46 (33%) | 0·068 (-0·01 – 0·22) |
| Number of aminophylline doses, mean (SD) | 8.4 (4.9) | 8.2 (5.6) | 0·855 (-1·83 – 2·20) |
| IV Phenobarbitone for seizures, N^o^ (%) | 2 (1%) | 1 (1%) | 0·574 (-0·02 – 0·03) |
| Multivitamins, N^o^ (%) | 72/140 (51%) | 72/137 (53%) | 0·851 (-0·13 – 0·11) |
| Folic acid, N^o^ (%) | 79 (56%) | 80 (58%) | 0·743 (-0·14 – 0·10) |
| Iron supplements, N^o^ (%) | 10 (7%) | 18 (13%) | 0·980 (-0·13 – 0·01) |

a. Significance level and 95% confidence intervals determined by 2-sample proportion test for categorical variables and 2-sample t test for continuous variables.

Abbreviations: bCPAP = bubble continuous positive airway pressure; CI = Confidence intervals; SD = Standard deviation

# Online-only figures

a. Criteria added or amended to increase relevance of aPBSI criteria to hospitalised neonates <2000g receiving KMC. Note – isolated apnoea refers to apneoa not temporally associated with milk aspiration or hypoglycemia.

Abbreviations: aPSBI= adapted Possible Serious Bacterial Infection; CPAP: Continuous positive airway pressure; h= hours; HR= heart rate; KMC= Kangaroo mother care; MRCG; Medical Research Council Unit The Gambia at LSHTM; NNU; Neonatal unit; RR=Respiratory rate; SpO_2_= Oxygen saturation

**eFigure 1. Overview of eligibility criteria, study procedures and key definitions for eKMC trial**

**
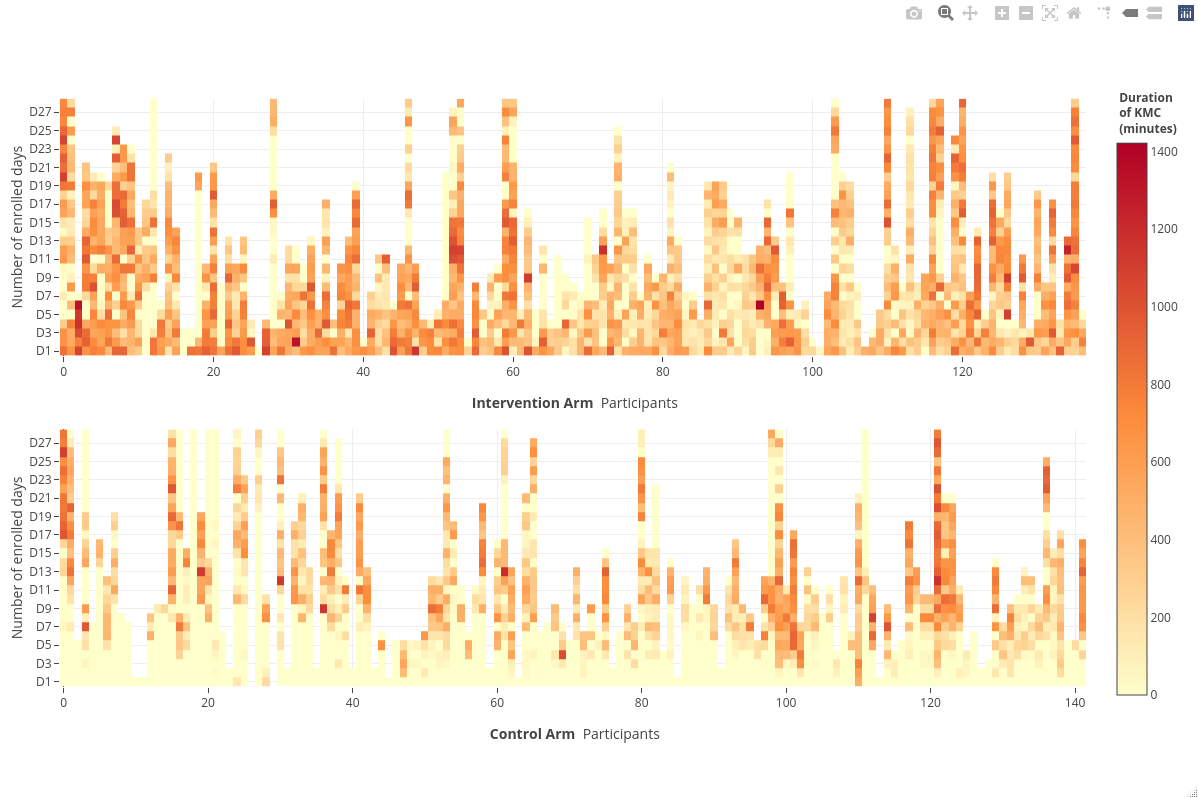
**

**eFigure 2. Duration (minutes) spent in kangaroo position, by allocation arm and day of enrolment**
